# Partial Replacement of Spray-Dried Blood Plasma with Alternative Animal and Insect Protein in Weaned Pig Diets: Effects on Growth Performance, Digestibility, and Gut Health

**DOI:** 10.3390/ani16193100

**Published:** 2026-10-02

**Authors:** Lucas M. Teixeira, Jansller L. Genova, Marcos E. Duarte, Jeferson P. Santana, Lais F. L. Reis, Anna L. M. Teixeira, Gabriel C. Rocha

**Affiliations:** 1 Muscle Biology and Nutrigenomics Laboratory, Department of Animal Science, Universidade Federal de Viçosa, Viçosa 36570-900, MG, Brazil; lucas.medina@ufv.br (L.M.T.); jansller.genova@ufv.br (J.L.G.); jeferson.santana@ufv.br (J.P.S.); lais.lino@ufv.br (L.F.L.R.); anna.l.teixeira@ufv.br (A.L.M.T.); 2 Phibro Animal Health Corporation, Mendon, IL 62351, USA; medzte@gmail.com

**Keywords:** alternative protein sources, gene expression, intestinal health, nutrient digestibility, spray-dried blood plasma, piglet performance

## Abstract

This study was to evaluate the effects of partially replacing spray-dried blood plasma (SDP) with alternative insect- and animal-derived protein meals on growth performance, diarrhea incidence (DI), nutrient digestibility, intestinal responses, and oxidative status of weaned pigs. A total of 135 pigs (21 d of age; 6.56 ± 0.79 kg body weight [BW]) were assigned to five treatments for 20 d. The control diet contained 40 g/kg SDP, whereas the other diets contained 20 g/kg SDP plus 20 g/kg black soldier fly larval meal (BSF), fish meal (FM), pork crackling meal (PCM), or hydrolyzed chicken protein (HCP). HCP maintained growth and energy and crude protein utilization comparable to SDP. PCM reduced final BW and average daily gain (ADG; *p* < 0.05), while average daily feed intake tended to decrease (*p* = 0.084). BSF and FM reduced DI (*p* < 0.05). BSF showed the lowest energy and crude protein digestibility (*p* < 0.05), whereas FM reduced crude protein digestibility and tended to reduce jejunal *PepT1* mRNA abundance (*p* = 0.052). BSF and PCM reduced jejunal *TNF-α* mRNA abundance, while FM and PCM reduced *ZO1* mRNA abundance (*p* < 0.05). Overall, HCP showed the greatest potential to partially replace SDP while preserving growth performance and nutrient utilization.

## Simple Summary

Spray-dried blood plasma is commonly used in diets for newly weaned pigs because of its nutritional and functional properties, but its relatively high cost encourages the search for alternative protein ingredients. This study evaluated whether black soldier fly larval meal, fish meal, pork crackling meal, or hydrolyzed chicken protein could partially replace spray-dried blood plasma in diets for weaned pigs. The protein sources differed in their effects on growth, nutrient utilization, and intestinal responses. Hydrolyzed chicken protein showed the greatest potential as a partial replacement because it maintained growth performance and nutrient utilization closer to those observed with spray-dried blood plasma. Black soldier fly larval meal and fish meal reduced diarrhea incidence, although black soldier fly larval meal showed lower nutrient digestibility. Pork crackling meal impaired piglet growth. Overall, these findings show that alternative protein ingredients should not be evaluated solely on their protein content, because their effects on nutrient utilization and intestinal health also determine their suitability for newly weaned pig diets.

## 1. Introduction

The early post-weaning period is a critical phase in pig production because piglets commonly experience reduced feed intake, impaired intestinal integrity, and increased susceptibility to enteric disorders, which together compromise nutrient utilization and growth performance [1,2]. In this context, post-weaning diets are typically formulated with palatable and highly digestible protein ingredients that can support intestinal adaptation and help mitigate the adverse effects of weaning. Among these, spray-dried blood plasma (SDP) has been widely used because of its recognized nutritional and functional value in nursery diets [3,4]. However, the relatively high cost of SDP can limit its use in commercial feeding programs [5,6].

As a result, there is increasing interest in alternative protein ingredients that are not only economically viable, but also capable of preserving, at least in part, the benefits associated with SDP during the nursery phase. This issue is particularly relevant because the practical value of a potential SDP replacer cannot be inferred from crude protein concentration alone. Rather, its suitability depends on a broader set of attributes, including digestibility, nutrient availability, palatability, and the capacity to support intestinal function and post-weaning adaptation in piglets.

In this regard, insect-derived ingredients and animal by-products have emerged as potential candidates for partial replacement of SDP. Black soldier fly larval meal (BSF; *Hermetia illucens*) has received considerable attention because it is rich in protein, essential amino acids, and other bioactive compounds that may contribute to piglet growth performance [7,8,9]. In addition, BSF contains medium-chain fatty acids and chitin, a structural polysaccharide of the larval exoskeleton, both of which have been associated with beneficial effects on intestinal health [7,10,11]. Lauric acid may exert antimicrobial activity [12,13], whereas chitin and its derivatives may have prebiotic properties [9,14]. On the other hand, BSF inclusion may also reduce nutrient digestibility, as chitin can form complexes with nutrients and interfere with digestive enzyme activity [15].

Fish meal (FM) is another relevant ingredient for nursery pig diets because of its high protein content, favorable amino acid profile, high digestibility, and contribution of minerals [4,16]. Beyond its nutritional value, FM has been associated with improved feed preference [17], enhanced growth performance, modulation of immune response (e.g., increasing immunoglobulin secretion while reducing pro-inflammatory cytokine production), and beneficial effects on intestinal morphology, microbiota composition, and nutrient digestibility [18,19].

Pork crackling meal (PCM), a by-product obtained directly from pig slaughter, is a protein-rich ingredient [20,21]. Its protein fraction is predominantly composed of collagen, whose hydrolysis can generate bioactive peptides with antioxidant activity and potential effects on the intestinal microbiota [22,23,24]. Nevertheless, PCM differs substantially from SDP in protein quality, because collagen-rich ingredients typically present an imbalanced amino acid profile and may not provide the same nutritional and functional contributions as SDP [25,26].

Hydrolyzed chicken protein (HCP) has also emerged as a promising ingredient in animal nutrition due to its high protein and energy content and its abundance of low-molecular-weight bioactive peptides and free amino acids generated during hydrolysis [4,27,28,29]. Protein hydrolysates derived from animal by-products have been reported to show high digestibility of protein, energy, and amino acids [28], in addition to influencing diet palatability and contributing to oxidative balance through free-radical scavenging activity [29,30,31,32]. These properties suggest that HCP may represent a feasible nutritional strategy in nursery diets, although its efficacy as a partial replacer of SDP remains insufficiently characterized.

Although previous studies support the use of some of these ingredients in nursery feeding programs, direct comparative evidence regarding their use as partial replacers of SDP is still limited. In particular, it remains unclear whether these ingredients can sustain piglet performance while also supporting nutrient utilization and intestinal health-related responses, and whether such responses differ according to the nutritional and functional characteristics of each protein source. This distinction is important because the value of replacing SDP in nursery diets depends not only on supplying protein, but also on maintaining digestive efficiency, intestinal environment, and post-weaning adaptation.

Therefore, we hypothesized that BSF, FM, PCM, and HCP would differ in their ability to partially replace SDP, particularly in their effects on growth performance and nutrient digestibility while supporting intestinal health in weaned pigs. To test this hypothesis, the objective of this study was to compare BSF, FM, PCM, and HCP as partial replacements for SDP in diets for weaned pigs, evaluating growth performance, diarrhea incidence, apparent total tract digestibility of energy and crude protein, intestinal pH and morphology, jejunal mRNA abundance of nutrient transporters, inflammatory and tight-junction markers, and serum indicators of oxidative status.

## 2. Materials and Methods

All methods involving the handling of pigs followed the ethical principles of animal research and were approved by the Commission of Ethics in the Use of Production Animals of Universidade Federal de Viçosa—UFV (protocol 013/2025).

### 2.1. Animals, Experimental Design, Housing and Diets

The experiment was conducted in an experimental facility at the Federal University of Viçosa, Minas Gerais, Brazil. One hundred and thirty-five piglets [PIC 337 (Large White × Landrace × Duroc × Pietrain) × Camborough (Large White × Landrace)], females and barrows in a similar proportion, were weaned at 20 d of age and with 6.56 ± 0.79 kg of body weight (BW). Piglets were allotted to a randomized complete block design based on initial BW, with three BW categories (light, medium, and heavy). Within each BW category, pigs were further organized into blocks, each consisting of five pens, and the five dietary treatments were manually and randomly assigned to the pens within each block. The design resulted in nine replicate pens per treatment, with three piglets per pen as the experimental unit.

The piglets were housed in suspended cages (0.54 m^2^/pig) equipped with semi-automatic feeders and nipple drinkers, with free access to feed and water. The cages were deliberately soiled with feces from sows from the same herd to increase microbial pressure [33]. The nursery room temperature was maintained at approximately 30 °C immediately after weaning and was gradually reduced by approximately 2 °C per week, reaching approximately 26 °C during the final week of the experimental period. Relative humidity was monitored throughout the experimental period and ranged from 59.7 ± 8.1% to 77.5 ± 8.6%. No vaccinations were administered during the experimental period. Piglets were monitored daily for general health status throughout the experiment. No health condition requiring therapeutic treatment or removal from the experiment was observed.

The piglets were fed according to a nursery feeding program in a single 21-d phase. The experimental treatments were composed of: (1) SDP, a control diet containing 40 g/kg spray-dried blood plasma (AP920, APC, Araçatuba, SP, Brazil); (2) BSF, in which 20 g/kg of SDP was replaced by 20 g/kg black soldier fly larval meal (*Hermetia illucens*; Cyns Pro, Cyns, Piracicaba, SP, Brazil); (3) FM, in which 20 g/kg of SDP was replaced by 20 g/kg fish meal (*Oreochromis niloticus*; MCassab, Rifaina, SP, Brazil); (4) PCM, in which 20 g/kg of SDP was replaced by 20 g/kg pork crackling meal (BRF Ingredients, Concórdia, SC, Brazil); and (5) HCP, in which 20 g/kg of SDP was replaced by 20 g/kg hydrolyzed chicken protein (Aminopac Bioaction, MCassab, Campo Grande, MS, Brazil). The animals had free access to feed and water during the experimental period. The experimental diets were formulated to be isoenergetic and isonitrogenous, meeting the nutritional requirements of pigs in the nursery phase, according to Rostagno et al. [4]. The substitution strategy was based on the inclusion of equal quantities of ingredients (*w*/*w*), and the experimental diets were formulated to be nutritionally comparable, considering the composition values of the ingredients adopted in the formulation (Table 1). The analyzed nutritional composition of the protein ingredients used in the experimental diets is presented in Table 2.

### 2.2. Growth Performance and Diarrhea Incidence

Throughout the trial, the offered diet and leftovers were weighed to calculate average daily feed intake (ADFI). At 21, 31, and 41 d of age, pigs were individually weighed to monitor BW, average daily gain (ADG), and calculate the feed conversion ratio (FCR). The fecal consistency of each pig was visually assessed by the same trained technician from 8:00 to 10:00 a.m. from 24 to 41 d of age using the method described by Teixeira et al. [34]. Scoring began at 24 d because feed intake is typically very low during the first days after weaning, limiting the expected dietary influence on fecal consistency. Fresh feces were scored on a four-point scale as follows: 0 = solid, 1 = semi-solid, 2 = semi- liquid, and 3 = liquid. Diarrhea incidence was defined as the occurrence of fecal consistency scores of 2 or 3 for 2 consecutive days. Diarrhea incidence was summarized at the pen level and expressed as a percentage, calculated as: (number of animals with diarrhea in each pen × days of diarrhea) ÷ (total number of animals in the pen × days observed) × 100%.

### 2.3. Fecal Collection, Feed Sampling and Apparent Total Tract Digestibility of Energy and Protein

Apparent digestibility was determined using the indicator method, in which Celite^®^ was included at a concentration of 10 g/kg diet. Pigs were fed the marked diets for a 5-d period (from 37 to 41 d of age) prior to and during fecal collection [35].

Fecal samples (200 g) were manually collected (from 7:00 a.m. to 4:00 p.m.) at 40 and 41 d of age from at least two pigs in each pen by rectal massage and stored in labeled aluminum containers. In addition, feed samples (100 g) from each of the five treatments were collected and stored in labeled polyethylene containers. Fecal samples collected from pigs in the same pen were grouped by pen before laboratory analysis. All fecal and feed samples were stored in a refrigerator at −20 °C until further analysis. For analysis, fecal samples were thawed at room temperature and manually homogenized, using disposable gloves, in a new, properly labeled aluminum container. Subsequently, fecal and experimental diet samples were weighed on an analytical balance (Bel Engineering, model M4102, Monza, Italy) and dried in a forced-air oven (Tecnal, model SF-325 NM, Piracicaba, SP, Brazil) at 55 °C for 72 h, as described by Detmann et al. [36]. After drying, fecal and feed samples were ground separately in a ball mill (Tecnal, model TE-350, Piracicaba, SP, Brazil) and stored in polyethylene containers.

To determine the concentration of the indicator in diets and feces, acid-insoluble residue was analyzed according to Method M-007/2 described by Detmann et al. [36]. The GE content of the diets and feces was measured using an adiabatic calorimeter bomb (Model 1356; Parr Instrument Company, Moline, IL, USA). The N content was determined by the Kjeldahl method (Method N-001/2), according to Detmann et al. [36]. The apparent total tract digestibility (ATTD) of gross energy (GE) and crude protein [CP; nitrogen (N) × 6.25] was calculated according to the equations of Adeola [37]. From these coefficients, the values of digestible protein (DP) and digestible energy (DE) were obtained by multiplying the respective coefficients by the dietary contents of CP or GE, with DP expressed as a percentage of the diet and DE expressed in kcal/kg of diet.

### 2.4. Blood Collection and Analysis

At 41 d of age, one piglet per pen, selected as the pig with BW closest to the pen average, was used for blood sampling and postmortem tissue collection. Briefly, blood was collected at 7:00 a.m. by orbital sinus puncture without anesthesia using a hypodermic needle (40 × 1.6 mm) into 10 mL tubes with clot activator (Cralplast, Cotia, SP, Brazil, glass vacuum tube). Immediately after collection, the blood was then centrifuged at 1800× *g* for 12 min to obtain serum, which was subsequently stored at −20 °C for oxidative status evaluation.

For antioxidant activity, catalase activity (CAT, U/mL) was determined according to Aebi [38] using H_2_O_2_ as substrate. Briefly, 1 mL of H_2_O_2_ was added to the reaction mixture containing 10 μL of sample in 1.0 mL of potassium phosphate buffer (50 mmol/L, pH 7.0). After addition, the reaction mixture was monitored at 240 nm for 1 min at an interval of 30 s. An extinction coefficient of ɛ240 = 0.036 mmol/L cm was used for calculations. One unit of CAT activity was defined as the amount of enzyme that decomposes one mmol H_2_O_2_ per min. The superoxide dismutase (SOD, U/mL) activity was assessed by the pyrogallol autoxidation method, based on the ability of this enzyme to catalyze the dismutation of superoxide (O_2_^−^) into O_2_ and H_2_O_2_ [39]. An aliquot of serum (10 μL) was incubated with 170 μL of sodium phosphate buffer (50 mM, pH 7.8) in a polystyrene microplate. The reaction was initiated by adding 20 μL of pyrogallol (100 μmol/L). The rate of increase in the absorbance at 320 nm was measured in a microplate spectrophotometer (Multiskan GO, Thermo Scientific, Waltham, MA, USA) after 30 min. One enzyme unit of SOD is defined as the amount that causes 50% inhibition of pyrogallol auto-oxidation.

The ferric reducing antioxidant power (FRAP, μmol/mL) was evaluated based on the reduction of Fe^3+^ to Fe^2+^ in the serum by nonenzymatic antioxidants and subsequent complexation of Fe^2+^ with 2,4,6-tri (2-pyridyl)-s-triazine to form the Fe^2+^ 2,4,6-tri (2-pyridyl)-s-triazine (2-pyridyl)-s-triazine chromophore. The maximum absorbance of the chromophores was measured spectrophotometrically at 595 nm [40].

Protein carbonyl (PC, nmol/mL) was determined according to Levine et al. [41] by reaction with 2,4-dinitrophenylhydrazine, with absorbance measured at 370 nm and quantification based on a molar extinction coefficient of ɛ370 = 22,000 mM/cm.

Nitric oxide (NO, nmol/mL) was indirectly determined from nitrite concentrations using the Griess method described by Guevara et al. [42]. Serum (50 μL) was reacted with 100 μL of Griess reagent, and absorbance was measured at 540 nm. NO levels were estimated using a sodium nitrite standard curve. Malondialdehyde (MDA, nmol/mL) was determined according to Jentzsch et al. [43]. Serum (10 μL) was reacted with trichloroacetic acid/thiobarbituric acid/hydrochloric acid solution and heated at 90 °C for 40 min. After cooling, butyl alcohol was added, followed by centrifugation at 9000× *g* for 10 min, and the absorbance of the upper phase was measured at 535 nm.

### 2.5. pH of Jejunum and Colon Contents

The same blood donor piglet was electrically stunned (240 V, 0.6 A for 3 s) followed by exsanguination to collect samples on day 42. Jejunal and colon contents were collected in sterile containers (50 mL) and, while still fresh, their pH was measured using a pH meter (Tec-3MP, Tecnal, Niort, France). The equipment was calibrated with standard buffer solutions of known pH (4.0 and 7.0), following the manufacturer’s recommendations. The electrode was washed with distilled water, and the pH was recalibrated between measurements [44].

### 2.6. Intestinal Morphology

Following exsanguination, the abdominal cavity was opened, the gastrointestinal tract was exposed, and the duodenum and jejunum were identified based on their anatomical location. Fragments measuring 2 cm were sampled (9 pigs/treatment) from the duodenum (10 cm from the pylorus junction) and jejunum (mid-section) for histological evaluation [45]. The histological sections were then washed in a physiological solution (0.9% sodium chloride) and fixed in 4% paraformaldehyde solution (100 mL 40% paraformaldehyde, 900 mL distilled water, 2.28 g monobasic sodium phosphate, and 21.74 g dibasic sodium phosphate) for 24 h at room temperature. After 24 h, duodenum and jejunum samples fixed in paraformaldehyde were transferred to a 70% ethanol solution [2].

Subsequently, the samples were sectioned into transverse fragments and dehydrated in increasing ethanol gradients, cleared in HistoChoice^®^ (Sigma-Aldrich, St. Louis, MO, USA), and embedded in liquid Paraplast^®^ (Leica Biosystems Inc., Buffalo Grove, IL, USA) at 65 °C. Slides were prepared with five transverse sections, each 5 μm thick. Sections were deparaffinized, rehydrated through a descending ethanol series, stained with hematoxylin and eosin (EasyPath, Indaiatuba, SP, Brazil), dehydrated through an ascending ethanol series, cleared, and mounted with coverslips. The sections were obtained in a semi-serial manner, selecting one out of every ten sections [2]. Morphological measurements (villus height and crypt depth) of the duodenum and jejunum were performed using an EVOS^™^ M5000 Cell Imaging System optical microscope (Invitrogen, Thermo Fisher Scientific, Waltham, MA, USA) with a 10× objective. ImageJ 1.50i software (Java 1.6.0_20; National Institutes of Health, Bethesda, MD, USA) was used for image analysis. Villus height (20 villi/slide) and crypt depth (20 crypts corresponding to the measured villi) were determined using the software. Only well-oriented villi and associated crypts were measured. The villus-to-crypt ratio was calculated from the length data. All analyses were performed by a single trained evaluator.

### 2.7. Relative mRNA Abundance

An additional 2 cm of jejunum was collected, immediately frozen in liquid N, and stored at −80 °C until total RNA extraction and subsequent gene expression analysis [45]. Total RNA was extracted as described by Correia et al. [2] and Kladt et al. [46]. For this, a commercial kit (SV Total RNA Isolation Kit-Promega, Z3100, Madison, WI, USA) was used, following the manufacturer’s instructions. Briefly, following extraction, RNA concentration was estimated using a NanoDrop^™^ Lite spectrophotometer (Thermo Fisher Scientific, Waltham, MA, USA). RNA integrity was assessed by 1.0% agarose gel electrophoresis. Complementary DNA (cDNA) was synthesized using the GoScript™ Reverse Transcription System (Promega Corporation, Madison, WI, USA, A2791). GenBank accession numbers (GenBank Overview [ncbi.nlm.nih.gov/]) were obtained and used to identify primers for the selected genes, which are listed in Table 3. The primers were applied for quantitative reverse transcription PCR (qRT-PCR), performed in duplicate, using GoTaq^®^ qPCR Master Mix (Promega, A6002) on a QuantStudio^®^ 3 system (Applied Biosystems, Thermo Fisher Scientific, Waltham, MA, USA). The thermal cycling conditions for the reactions followed the manufacturer’s instructions for the GoTaq^®^ qPCR Master Mix kit (Promega Corporation, Madison, WI, USA) and consisted of: polymerase activation at 95 °C for 2 min, followed by 40 denaturation cycles at 95 °C for 15 s and annealing and extension at 60 °C for 1 min. For normalization of target gene expression in jejunal samples, the Ct values of the beta-2-microglobulin (*B2M*) gene were used due to its stable expression in piglet intestinal tissues, as described by Wang et al. [47]. Relative gene expression was quantified using the ΔCt calculation method [48]. The SDP group was used as the reference group (calibrator), and its expression level was set to 1.0. The target genes analyzed were: glutathione peroxidase (*GPX*), superoxide dismutase (*SOD*), catalase (*CAT*), occludin (*OCL*), zonula occludens 1 (*ZO1*), interferon gamma (*IFN-γ*), tumor necrosis factor alpha (*TNF-α*), interleukin 10 (*IL-10*), glucose transporter type 2 (*GLUT2*), sodium/glucose cotransporter 1 (*SGLT1*), monocarboxylate transporter 1 (*MCT1*), peptide transporter 1 (*PepT1*), excitatory amino acid carrier 1 (*EAAC1*), and Na+-independent cationic and Na+-dependent neutral amino acid transporter 1 (*y+LAT1*).

### 2.8. Statistical Procedures

All analyses were performed in SAS 9.4 (Statistical Analysis System Inc., Cary, NC, USA), using PROC MEANS for descriptive statistics and PROC GLIMMIX for inference. Observations with |studentized residual| > 2.5 were considered outliers; however, no outliers were detected, and all observations were retained in the analyses. Model adequacy was assessed from studentized residuals (normal Q–Q plots and Shapiro–Wilk). For each response variable, observations treated as continuous were modeled using linear mixed models with a Gaussian distribution and identity link. Treatment was included as a fixed effect and block as a random factor. Residuals were assumed independent and homoscedastic within blocks. Parameters were estimated by restricted maximum likelihood. Denominator degrees of freedom for tests of fixed effects were obtained using the Kenward–Roger method. For each response, overall treatment effects were tested by Type III F-tests (α = 0.05). Diarrhea incidence was summarized at the pen level as a percentage over the observation period and analyzed as a continuous response variable using a linear mixed model. When the main effect was significant, pairwise comparisons among treatments were adjusted using Tukey–Kramer. Least-squares means with pooled standard errors were reported. When relevant, tendencies were declared for 0.05 < *p* ≤ 0.10.

## 3. Results

### 3.1. Growth Performance and Diarrhea Incidence

From 21 to 31 d of age, final BW (*p* = 0.085), ADG (*p* = 0.090), and FCR (*p* = 0.086) tended to differ among treatments (Table 4). Pork crackling meal resulted in lower BW and ADG than SDP, and poorer FCR than SDP and FM, whereas BSF, FM, and HCP showed intermediate values for final BW and ADG. ADFI was not affected (*p* > 0.10). From 31 to 41 d, final BW was lower in PCM-fed pigs (*p* = 0.041), while ADG, ADFI, and FCR remained unaffected (*p >* 0.10). Across the entire period (21 to 41 d), ADG was lower in PCM-fed pigs than in those fed SDP, BSF, FM, and HCP (*p* = 0.042). ADFI tended (*p* = 0.084) to be lower in PCM-fed pigs, with SDP and HCP showing the highest ADFI and BSF and FM showing intermediate values. FCR did not differ among treatments. Diarrhea incidence was significantly affected (*p* < 0.001), with BSF and FM markedly reducing DI compared with SDP, while PCM and HCP presented intermediate responses.

### 3.2. Apparent Total Tract Digestibility of Energy and Protein

Pigs fed SDP, PCM, and HCP had higher DE (*p* < 0.001) than those fed BSF or FM, with BSF showing the lowest value (Table 5). For ATTD of GE, pigs fed SDP had a higher value (*p* < 0.001) than those fed BSF, FM, and PCM, whereas BSF showed the lowest coefficient. HCP showed an intermediate value. For DP, pigs fed SDP had a higher value (*p* < 0.001) than those fed BSF and FM, whereas PCM and HCP showed intermediate values and differed only from FM. In addition, SDP showed a higher ATTD of CP (*p* < 0.001) than BSF, FM, and PCM, whereas HCP showed an intermediate value. BSF showed the lowest ATTD of CP.

### 3.3. Antioxidant Activity and Oxidative/Nitrosative Damage

Dietary treatments did not affect antioxidant activity or markers of oxidative/nitrosative damage, as indicated by NO, CAT, MDA, SOD, FRAP, and PC (*p* > 0.10; Table 6).

### 3.4. Intestinal Morphology and pH of Jejunum and Colon Contents

Jejunal crypt depth tended to be affected by dietary treatments (*p* = 0.083), with BSF showing a lower value than SDP, whereas FM, PCM, and HCP showed intermediate values (Table 7). No effects of dietary treatments were observed on villus height or villus:crypt ratio in the jejunum, or on villus height, crypt depth, or villus:crypt ratio in the duodenum (*p* > 0.10). Jejunal and colonic pH were also unaffected by dietary treatments (*p* > 0.10).

### 3.5. mRNA Relative Abundance of Inflammatory, Antioxidant, Nutrient Transporter, and Tight-Junction Markers

Pigs fed FM and PCM showed lower jejunal *ZO1* mRNA abundance (*p* = 0.049; Table 8) than those fed SDP, whereas BSF and HCP showed intermediate values. Pigs fed BSF and PCM showed lower *TNF-α* mRNA abundance (*p* = 0.022) than those fed SDP, whereas FM and HCP showed intermediate values. *PepT1* mRNA abundance tended to be lower (*p* = 0.052) in pigs fed FM than in those fed SDP, whereas BSF, PCM, and HCP showed intermediate values. No effects of dietary treatments were observed on the mRNA abundance of *CAT*, *GPX*, *SOD*, *OCL*, *EAAC1*, *GLUT2*, *IL-10*, *IFN-γ*, *MCT1*, *SGLT1*, or *y+LAT1* (*p* > 0.10).

## 4. Discussion

The present study demonstrated that alternative protein ingredients differed in their ability to partially replace SDP in diets for weaned pigs, providing partial support for our initial hypothesis. HCP more closely preserved growth performance and nutrient digestibility relative to SDP, whereas BSF and FM reduced diarrhea incidence despite showing lower nutrient digestibility. In contrast, PCM impaired growth performance, despite reducing jejunal TNF-α mRNA abundance. These findings indicate that the suitability of alternative protein ingredients as partial replacements for SDP depends on ingredient-specific nutritional and functional characteristics, rather than on their crude protein contribution alone.

The poorer growth performance in pigs fed PCM may be explained, at least in part, by the collagen-rich amino acid profile of this ingredient, which may have reduced the biological value of the absorbed protein and altered the balance between indispensable and dispensable amino acids available to the pigs. Specifically, the thermal processing applied during PCM manufacturing may induce heat damage to heat-sensitive indispensable amino acids (such as lysine), reducing their true ileal availability [49,50]. In addition, the characteristic collagen-derived peptide profile, coupled with a disproportionate excess of glycine and proline [25,26], may contribute to an imbalanced amino acid supply, thereby increasing amino acid catabolism and N excretion [51]. This poorer growth response was accompanied by lower crude protein utilization, supporting the interpretation that PCM protein, despite its high crude protein content, was used less efficiently by the pigs.

In the present study, pigs fed BSF and FM showed lower DI than those fed SDP, which was an unexpected response because partial or total replacement of SDP is more often associated with increased DI after weaning. Indeed, reports of reduced diarrhea after SDP withdrawal are limited [52,53], whereas most studies describe a higher DI under this condition [5,54,55,56]. The lower DI observed in BSF- and FM-fed pigs suggests that these ingredients may have influenced post-weaning intestinal stability through multiple, non-mutually exclusive mechanisms. Because BSF and FM showed lower crude protein digestibility than SDP, the reduction in DI was unlikely to be explained by improved protein digestion per se, suggesting that ingredient-specific effects on intestinal microbial activity, fermentation patterns, or mucosal responses may have contributed to this response [34,57,58]. From a microbiological perspective, BSF may also provide bioactive compounds, including chitin and specific fatty acids, with antimicrobial activity and microbiota-modulating potential [12,13,59]. In addition, FM has been associated with improvements in local immune responses and intestinal microbial ecology in piglets [19]. Collectively, these effects may have contributed to the lower risk of enteric disturbance observed in the BSF and FM groups. Supporting this interpretation, the lower DI observed in BSF-fed pigs was accompanied by reduced jejunal *TNF-α* mRNA abundance, whereas PCM also decreased *TNF-α* mRNA abundance relative to SDP. Because *TNF-α* is a key pro-inflammatory cytokine associated with epithelial dysfunction and tight-junction disorganization, lower mRNA abundance of this marker may reflect a less activated inflammatory state in the intestinal mucosa [60,61]. In the BSF group, this response was also accompanied by a tendency toward lower jejunal crypt depth compared with SDP, which may indicate reduced epithelial turnover and proliferative demand in the intestinal mucosa [62]. Thus, at least for BSF, the reduction in DI was accompanied by signals consistent with improved intestinal stability.

Although FM and PCM reduced *ZO1* mRNA abundance, with no change in *OCL* mRNA abundance, these responses were not accompanied by alterations in intestinal morphology or systemic oxidative status. Therefore, these findings should be interpreted cautiously. Changes in mRNA abundance of tight-junction-related genes do not necessarily translate into functional changes in epithelial permeability, particularly in the absence of direct barrier measurements, and may instead reflect modest transcriptional adjustments without overt impairment of barrier integrity [63]. Overall, the present results do not provide evidence of marked intestinal structural damage in the FM and PCM groups. However, for PCM, this interpretation must be tempered by the concomitant poorer growth performance, suggesting that limitations associated with protein quality may have constrained the biological response despite the lower *TNF-α* mRNA abundance [1,64,65,66].

In addition to inflammatory and morphological responses, changes in intestinal nutrient transporters may help explain part of the ingredient-specific effects observed in the present study. *PepT1* is the main transporter responsible for the absorption of dietary di- and tripeptides in the small intestine of pigs, and its expression can be modulated by diet composition and by the form in which N is delivered to the intestinal lumen, including peptide size and the relative contribution of free amino acids [67,68,69]. In the present study, FM tended to reduce jejunal *PepT1* mRNA abundance relative to SDP, and this response occurred together with lower ATTD of CP and DP. Together, these findings suggest that FM may have altered the luminal profile or timing of peptide release along the small intestine, which in turn may have contributed to the lower jejunal *PepT1* mRNA abundance. This interpretation is biologically plausible because *PepT1* mRNA abundance is known to respond to substrate availability in the intestinal lumen [70,71,72]. Although luminal peptide profiles were not determined in the present study, previous evidence indicates that the source and digestion kinetics of dietary protein can influence the site and rate of peptide and amino acid release along the small intestine [27], which is consistent with the hypothesis that FM modified the dynamics of peptide availability along the intestine.

Intestinal responses in weaned pigs can be influenced by multiple factors, including environmental conditions, microbial challenge, and dietary composition [73]. In the present study, however, all dietary treatments were subjected to the same housing conditions and microbial pressure. Therefore, although these factors may have contributed to the overall intestinal challenge experienced by the pigs, the differences observed among treatments in *ZO1* and *TNF-α* mRNA abundance, as well as the tendency observed for *PepT1*, are more likely associated with the specific characteristics of the dietary protein sources.

From a nutritional perspective, the ATTD of GE and CP, as well as DE and DP values, are useful indicators of the effective nutritional value of feed ingredients because they reflect how efficiently dietary nutrients are released, digested, and made available to the animal along the gastrointestinal tract [74]. Our initial hypothesis was that partially replacing SDP with insect- and animal-derived protein sources would provide an adequate nutrient supply to support piglet performance. However, the present results indicate that nutrient utilization differed markedly among ingredients. SDP showed the most favorable digestibility responses, whereas BSF consistently showed the lowest coefficients for both energy and protein utilization. Among the alternative ingredients, HCP and PCM yielded DE values closer to SDP, whereas FM was intermediate. However, although PCM showed DE values similar to SDP, its ATTD of GE was lower, while HCP was the only alternative that did not differ from SDP for this coefficient. These results suggest that HCP has greater potential to replace SDP with less impact on dietary energy utilization [75]. Although the diets were formulated to be isoenergetic and isonitrogenous and to meet the nutritional requirements of the animals, the observed differences among treatments indicate that the effective nutritional value of the diets depended on ingredient-specific characteristics that were not fully captured by the theoretical formulation values alone.

This discrepancy between formulated composition and biological response was particularly evident for PCM. In the present study, pigs fed PCM showed poorer growth performance even though this treatment did not present the lowest digestibility coefficients. This pattern suggests that the productive limitation associated with PCM was not explained solely by the overall extent of digestion, but more likely by the biological quality of the absorbed protein. In this context, collagen-rich ingredients may reduce the efficiency of protein utilization because of their unbalanced amino acid profile, characterized by lower concentrations of indispensable amino acids and a greater relative contribution of dispensable amino acids [25,26], which may limit N utilization efficiency and thereby impair growth. Because ileal amino acid digestibility and N retention were not measured in the present study, this interpretation should be viewed as a biologically plausible hypothesis rather than a definitive mechanism. Nevertheless, given the scarcity of studies evaluating PCM in nursery pig diets, this possibility deserves further investigation.

In contrast, the reduction in energy and CP digestibility observed in BSF-fed pigs indicates an important nutritional limitation of this ingredient under the conditions of the present study. This finding contrasts with some reports in weaned pigs, in which BSF inclusion did not impair energy digestibility, although CP digestibility has shown greater variability depending on ingredient processing and composition [9,76,77]. These discrepancies may be related to inclusion level, insect developmental stage, processing conditions, animal age, and especially the chitin content of the BSF meal [8,10,12,78]. Chitin, a structural polysaccharide present in the insect exoskeleton, is poorly degraded by pigs and may reduce nutrient utilization by limiting enzyme access to lipids and proteins [15,79,80]. In the present study, the BSF meal contained 3.11% chitin; however, because BSF was included at only 20 g/kg of diet, its contribution corresponded to approximately 0.62 g chitin/kg of diet. Therefore, although chitin may have contributed to the lower nutrient digestibility, its relatively low dietary concentration suggests that it is unlikely to fully explain the magnitude of the reduction observed. Other ingredient-specific characteristics, including processing-related changes and the structural organization of nutrients within the BSF meal, may also have limited nutrient accessibility. In agreement with the potential contribution of ingredient structure, Bonomini et al. [81] reported markedly lower protein solubilization in chitin-rich BSF fractions than in fractions depleted of this polymer. Thus, the present results suggest that the nutritional value of BSF cannot be inferred solely from its CP concentration, because ingredient composition and structure may substantially influence the effective release and digestion of nutrients.

Finally, from a sustainability standpoint, ingredients with lower protein utilization efficiency may increase N losses to the environment by increasing the fraction of dietary N that is not effectively retained by the animal [34,82]. Although N excretion was not directly measured in the present study, this perspective is relevant when interpreting alternative protein sources for nursery diets. In this context, HCP showed the greatest potential as a partial substitute for SDP under the conditions evaluated here. In contrast, BSF showed potentially beneficial effects on DI and inflammatory signaling; however, its lower digestibility represents an important nutritional limitation that may also reduce nutrient use efficiency. Therefore, further research is needed to identify the ingredient-specific factors responsible for this lower digestibility and to evaluate processing or nutritional strategies capable of improving nutrient accessibility. Although chitin-degrading enzymes may represent one such strategy, particularly because chitin can limit nutrient accessibility, the relatively low dietary contribution of chitin in the present study suggests that other compositional and processing-related factors should also be considered. In this regard, Yang et al. [15] reported that chitinase supplementation improved energy and CP digestibility and increased ileal digestibility of several amino acids in pigs. Future studies should therefore investigate strategies targeting both chitin and other factors limiting nutrient utilization to better define the nutritional potential of BSF in nursery pig diets.

## 5. Conclusions

Hydrolyzed chicken protein showed the greatest potential to partially replace SDP, as it maintained growth performance and nutrient utilization closer to those observed with SDP. In contrast, PCM impaired growth performance, while BSF and FM reduced diarrhea incidence despite their lower nutrient utilization. These findings indicate that the suitability of alternative protein sources for weaned pigs depends not only on their protein contribution but also on their effects on nutrient utilization and intestinal responses. Therefore, HCP may represent a practical alternative for partial replacement of SDP in nursery diets, whereas the use of the other evaluated protein sources should consider their specific nutritional and physiological effects.

## Figures and Tables

**Table 1:** Ingredients and chemical composition of diets fed to nursery pigs (g/kg, as fed basis).

Ingredients	Treatments ^1^				
	SDP	BSF	FM	PCM	HCP
Ground corn, 7.8% CP	527.2	518.0	530.0	530.8	527.9
Soybean meal, 46.0% CP	237.3	244.4	237.5	231.4	233.3
Dried whey, 12.5% CP	110.0	110.0	110.0	110.0	110.0
Plasma protein, 78.0% CP	40.0	20.0	20.0	20.0	20.0
Black soldier fly, 53.0% CP	-	20.0	-	-	-
Fish meal, 65.0% CP	-	-	20.0	-	-
Pork crackling meal, 80.0% CP	-	-	-	20.0	-
Hydrolyzed chicken protein, 77.2% CP	-	-	-	-	20.0
Sugar	30.0	30.0	30.0	30.0	30.0
Bicalcium phosphate	13.1	13.6	11.5	14.2	13.5
Calcitic limestone	8.7	6.7	5.6	8.2	8.4
Soybean oil	18.7	18.7	17.3	16.9	19.0
Salt	0.5	1.8	1.7	1.8	1.7
L-lys, 78.0%	5.0	5.7	5.6	5.8	5.7
DL-met, 99.0%	2.8	2.9	2.9	2.9	3.0
L-thr, 98.5%	2.6	3.0	3.0	3.1	3.0
L-val, 96.5%	1.5	1.8	1.8	1.7	1.8
L-trp, 99.0%	0.7	0.9	0.9	0.9	0.8
L-ile, 98.0%	0.6	0.7	0.6	0.6	0.6
L-his, 98.0%	-	0.1	0.2	0.3	-
Vitamin-mineral premix ^2^	1.4	1.4	1.4	1.4	1.4
Calculated composition ^3^					
ME, kcal/kg	3400	3400	3400	3400	3400
Crude protein, %	20.5	20.5	20.5	20.6	20.5
SID ^4^ lys, %	1.45	1.45	1.45	1.45	1.45
SID met, %	0.53	0.56	0.56	0.55	0.56
SID met + cys, %	0.87	0.87	0.87	0.87	0.87
SID thr, %	0.98	0.98	0.98	0.98	0.98
SID trp, %	0.30	0.30	0.30	0.30	0.30
SID val, %	1.00	1.00	1.00	1.00	1.00
SID arg, %	1.13	1.12	1.12	1.13	1.13
SID ile, %	0.80	0.80	0.80	0.80	0.80
Total Ca, %	0.70	0.70	0.70	0.70	0.70
Available P, %	0.49	0.49	0.49	0.49	0.49
Total Na, %	0.25	0.25	0.25	0.25	0.25
Crude fiber, %	1.96	1.97	1.96	1.94	1.94
Lactose, %	8.47	8.47	8.47	8.47	8.47

^1^ Dietary treatment: SDP, a control diet containing 40 g/kg spray-dried blood plasma; BSF, in which 20 g/kg of SDP was replaced by 20 g/kg black soldier fly larval meal; FM, in which 20 g/kg of SDP was replaced by 20 g/kg fish meal; PCM, in which 20 g/kg of SDP was replaced by 20 g/kg pork crackling meal; HCP, in which 20 g/kg of SDP was replaced by 20 g/kg hydrolyzed chicken protein. ^2^ Mineral and vitamin premix provided the following per kilogram of complete diet: copper: 10 mg; iodine: 1.5 mg; iron: 100 mg; manganese: 40 mg; selenium: 0.3 mg; zinc: 100 mg; vitamin A: 12,000 IU; vitamin D3: 2250 IU; vitamin E: 65 IU; vitamin K: 3 mg; thiamine: 2.25; riboflavin: 6 mg; pyridoxine: 2.25 mg; vitamin B12: 27 mcg; folic acid: 400 mcg; biotin: 150 mcg; pantothenic acid: 22.5 mg; niacin: 45 mg. ^3^ Calculated composition of dietary ingredients based on Rostagno et al. [4]. ^4^ Standardized ileal digestible.

**Table 2:** Analysis of the nutritional composition of highly digestible protein sources used in diets for pigs from 21 to 41 d of age (as-fed basis).

Nutritional Contents	Protein Ingredients ^1^				
	SDP	BSF	FM	PCM	HCP
GE ^2^, kcal/kg	4433	5059	4294	5407	4864
CP ^2^, %	79.46	54.18	62.65	82.81	74.25
Chitin, %	-	3.11	-	-	-
EE ^2^, %	0.45	12.06	10.57	14.29	4.91
Ash, %	8.60	7.98	20.77	2.77	6.35
Ca, %	0.60	0.70	6.47	0.13	0.73
P, %	0.86	1.10	3.51	0.39	0.76
K, %	0.18	1.61	0.93	0.59	1.12
Mg, %	0.01	0.34	1.15	0.04	0.08
Dry matter, %	92.80	93.54	94.06	94.04	95.00
Indispensable amino acids, %					
Histidine	2.55	1.40	1.22	1.62	1.49
Isoleucine	2.82	2.31	2.21	2.13	3.12
Leucine	7.76	3.60	3.81	4.14	5.22
Lysine	7.36	3.11	3.73	4.16	5.33
Methionine	1.05	0.92	1.54	1.28	1.79
Phenylalanine	4.42	2.08	2.08	2.32	2.79
Threonine	5.30	2.11	2.50	2.26	3.18
Tryptophan	0.98	0.58	0.39	0.40	0.66
Tyrosine	4.07	2.93	1.55	1.63	2.32
Valine	5.46	3.03	2.53	2.99	3.57
Dispensable amino acids, %					
Alanine	4.20	3.58	4.32	6.11	4.56
Arginine	4.64	2.59	3.98	5.84	4.87
Aspartic acid	9.37	5.37	4.58	6.53	7.06
Cysteine	2.33	0.42	0.38	0.44	0.62
Glycine	2.98	3.19	6.93	13.31	5.95
Glutamic acid	11.00	6.40	7.15	9.49	9.98
Hydroxyproline	<0.01	<0.01	1.96	4.80	1.46
Proline	4.32	2.93	4.19	8.18	4.07
Serine	5.27	2.34	2.46	3.04	3.21
Taurine	<0.01	<0.01	0.52	<0.01	0.54
Total amino acids	84.91	48.31	57.65	80.25	71.13

^1^ Dietary treatment: SDP, a control diet containing 40 g/kg spray-dried blood plasma; BSF, in which 20 g/kg of SDP was replaced by 20 g/kg black soldier fly larval meal; FM, in which 20 g/kg of SDP was replaced by 20 g/kg fish meal; PCM, in which 20 g/kg of SDP was replaced by 20 g/kg pork crackling meal; HCP, in which 20 g/kg of SDP was replaced by 20 g/kg hydrolyzed chicken protein. ^2^ GE = gross energy; CP = crude protein; EE = ether extract.

**Table 3:** List of primers used in reverse transcription quantitative PCR gene expression analysis in weaned pigs.

Genes ^1^	GenBank Number	Sequence ^2^
Target genes		
*GPX*	NM_214201.1	F: 5′-GCCCAACTTCATGCTCTTC-3′ R: 5′-CAGGATCTCCCATTCTTGGC-3′
*SOD*	NM_001190422.1	F: 5′-ATCAAGAGAGGCACGTTGGA-3′ R: 5′-TCTGCCCAAGTCATCTGGTT-3′
*CAT*	NM_214301.2	F: 5′-GCTTTAGTGCTCCCGAACAG-3′ R: 5′-AGATGACCCGCAATGTTCTC-3′
*OCL*	NM_001163647.1	F: 5′-TCCTGGGTGTGATGGTGTTC-3′ R: 5′-CGTAGAGTCCAGTCACCGCA-3′
*ZO1*	XM_003353439.2	F: 5′-AAGCCCTAAGTTCAATCACAATCT-3′ R: 5′-ATCAAACTCAGGAGGCGGC-3′
*IFN-γ*	NM_213948	F: 5′-TGGTAGCTCTGGGAAACTGAATG-3′ R: 5′-GGCTTTGCGCTGGATCTG-3′
*TNF-α*	NM_214022.1	F: 5′-CATCGCCGTCTCCTACCA-3′ R: 5′-CCCAGATTCAGCAAAGTCCA-3′
*IL-10*	NM_214041.1	F: 5′-GAAGGACCAGATGGGCGACTT-3′ R: 5′-CACCTCCTCCACGGCCCTTG-3′
*GLUT2*	FJ209732.1	F: 5′- TTGCCTTGGATGAGTTATGTGA-3′ R: 5′-GCGTGGTCCTTGACTGAAAA-3′
*SGLT1*	MW_280290.1	F: 5′-GGTTGGAGCTTCTCTGTTTG-3′ R: 5′-CCAGAACAACCACCCAAATC-3′
*MCT1*	AM_286425.1	F: 5′-GGTGGAGGTCCTATCAGCAG-3′ R: 5′-AAGCAGCCGCCAATAATCAT-3′
*PepT1*	AY_180903.1	F: 5′-CATGGATGCTGTGTGTATC-3′ R: 5′-CATGGAAGCCAGGAACATC-3′
*EAAC1*	NM_001164649.1	F: 5′-CAGTGGATGCCATGTTAGAC-3′ R: 5′-CGTCTCTGGCTCACTAGAA-3′
*y+LAT1*	EU_047705.1	F: 5′-TGC GTG GAG GAC ATCTT-3′ R: 5′-CTC CTT CCA GCG CAA ATA G-3′
Normalizing gene		
*B2M*	NM_213978.1	F: 5′TGAAAAACGGGGAGAAGATG3′ R: 5′GTGATGCCGGTTAGTGGTCT3′

^1^ *GPX* = glutathione peroxidase; *SOD* = superoxide dismutase; *CAT* = catalase; *OCL* = occludin; *ZO1* = zonula occludens 1; *IFN-γ* = interferon gamma; *TNF-α* = tumor necrosis factor alpha; *IL-10* = interleukin 10; *GLUT2* = glucose transporter type 2; *SGLT1* = Na+/glucose co-transporter 1; *MCT1* = monocarboxylate transporter 1; *PepT1* = peptide transporter 1; *EAAC1* = excitatory amino acid carrier 1; *y+LAT1* = Na+-independent cationic and Na+-dependent neutral amino acid transporter 1; *B2M* = beta-2-microglobulin. ^2^ F and R indicate Forward and Reverse primers, respectively.

**Table 4:** Effect of insect- and animal-derived protein ingredients on the performance and diarrhea incidence (DI) of pigs from 21 to 41 d of age.

Item ^4^	Dietary Treatments ^3^					SEM ^1^	*p*-Value ^2^
	SDP	BSF	FM	PCM	HCP		
Initial BW, kg	6.57	6.56	6.56	6.57	6.57	0.527	0.994
21 to 31 d							
Final BW, kg	8.77 ^A^	8.47 ^AB^	8.58 ^AB^	8.24 ^B^	8.60 ^AB^	0.538	0.085
ADG, kg	0.22 ^A^	0.19 ^AB^	0.20 ^AB^	0.17 ^B^	0.20 ^AB^	0.013	0.090
ADFI, kg	0.28	0.26	0.26	0.24	0.27	0.014	0.323
FCR, kg:kg	1.30 ^B^	1.39 ^AB^	1.30 ^B^	1.45 ^A^	1.34 ^AB^	0.042	0.086
31 to 41 d							
Final BW, kg	13.02 ^a^	12.84 ^a^	12.81 ^a^	12.02 ^b^	12.93 ^a^	0.909	0.041
ADG, kg	0.43	0.44	0.42	0.38	0.43	0.046	0.333
ADFI, kg	0.66	0.59	0.57	0.52	0.60	0.065	0.218
FCR, kg:kg	1.59	1.36	1.35	1.38	1.38	0.072	0.140
21 to 41 d							
ADG, kg	0.32 ^a^	0.31 ^a^	0.31 ^a^	0.27 ^b^	0.32 ^a^	0.020	0.042
ADFI, kg	0.47 ^A^	0.42 ^AB^	0.42 ^AB^	0.38 ^B^	0.43 ^A^	0.036	0.084
FCR, kg:kg	1.47	1.35	1.34	1.40	1.36	0.047	0.287
DI, %	51.85 ^a^	22.22 ^b^	31.34 ^b^	38.46 ^ab^	35.62 ^ab^	4.514	<0.001

^a,b^ Means followed by different lowercase letters differ statistically at the *p* ≤ 0.05 level, while uppercase letters were used to indicate a trend for 0.05 < *p* < 0.10. ^1^ Pooled standard error of the mean; *n* = 9 pens per treatment. ^2^ *p*-value referring to ANOVA. ^3^ Dietary treatment: SDP, a control diet containing 40 g/kg spray-dried blood plasma; BSF, in which 20 g/kg of SDP was replaced by 20 g/kg black soldier fly larval meal; FM, in which 20 g/kg of SDP was replaced by 20 g/kg fish meal; PCM, in which 20 g/kg of SDP was replaced by 20 g/kg pork crackling meal; HCP, in which 20 g/kg of SDP was replaced by 20 g/kg hydrolyzed chicken protein. ^4^ DI = diarrhea incidence was considered to be the presence of feces with a score of 2 or 3 for two consecutive days; BW = body weight; Initial BW = initial body weight; Final BW = body weight; ADG = average daily gain; ADFI = average daily feed intake; FCR = feed conversion ratio.

**Table 5:** Effect of insect- and animal-derived protein ingredients on the apparent total tract digestibility of energy and protein of pigs at 41 d of age.

Item ^4^	Dietary Treatments ^3^					SEM ^1^	*p*-Value ^2^
	SDP	BSF	FM	PCM	HCP		
DE, kcal/kg	3883 ^a^	3419 ^c^	3583 ^b^	3763 ^a^	3781 ^a^	31.650	<0.001
ATTD of GE, %	86.16 ^a^	77.65 ^c^	82.32 ^b^	82.33 ^b^	84.40 ^ab^	0.715	<0.001
DP, %	16.94 ^a^	15.77 ^bc^	14.68 ^c^	15.98 ^ab^	16.02 ^ab^	0.275	<0.001
ATTD of CP, %	79.92 ^a^	71.38 ^c^	74.18 ^bc^	74.34 ^bc^	77.42 ^ab^	1.319	<0.001

^a,b,c^ Means followed by different lowercase letters differ statistically at the *p* ≤ 0.05 level. ^1^ Pooled standard error of the mean; *n* = 9 pens per treatment. ^2^ *p*-value referring to ANOVA. ^3^ Dietary treatment: SDP, a control diet containing 40 g/kg spray-dried blood plasma; BSF, in which 20 g/kg of SDP was replaced by 20 g/kg black soldier fly larval meal; FM, in which 20 g/kg of SDP was replaced by 20 g/kg fish meal; PCM, in which 20 g/kg of SDP was replaced by 20 g/kg pork crackling meal; HCP, in which 20 g/kg of SDP was replaced by 20 g/kg hydrolyzed chicken protein. ^4^ DE = digestible energy; ATTD of GE = apparent total tract digestibility of gross energy; DP = digestible protein; ATTD of CP = apparent total tract digestibility of crude protein.

**Table 6:** Effect of insect- and animal-derived protein ingredients on antioxidant activity and oxidative/nitrosative damage of pigs at 41 d of age.

Item ^4^	Dietary Treatments ^3^					SEM ^1^	*p*-Value ^2^
	SDP	BSF	FM	PCM	HCP		
NO, nmol/mL	198.57	187.05	190.29	214.12	207.13	18.989	0.612
CAT, U/mL	78.37	83.66	84.28	81.56	76.43	7.681	0.939
MDA, nmol/mL	5.34	5.48	5.11	4.81	5.23	0.301	0.241
SOD, U/mL	4.19	4.23	5.02	4.75	4.19	0.359	0.342
FRAP, μmol/mL	0.26	0.27	0.25	0.27	0.23	0.013	0.208
PC, nmol/mL	0.47	0.54	0.50	0.44	0.46	0.047	0.619

^1^ Pooled standard error of the mean; *n* = 9 pigs per treatment. ^2^ *p*-value refers to ANOVA. ^3^ Dietary treatment: SDP, a control diet containing 40 g/kg spray-dried blood plasma; BSF, in which 20 g/kg of SDP was replaced by 20 g/kg black soldier fly larval meal; FM, in which 20 g/kg of SDP was replaced by 20 g/kg fish meal; PCM, in which 20 g/kg of SDP was replaced by 20 g/kg pork crackling meal; HCP, in which 20 g/kg of SDP was replaced by 20 g/kg hydrolyzed chicken protein. ^4^ FRAP = ferric reducing antioxidant power; CAT = catalase; SOD = superoxide dismutase; PC = protein carbonyl; NO = nitric oxide; MDA = malondialdehyde.

**Table 7:** Effect of insect- and animal-derived protein ingredients on intestinal morphology and pH of jejunal and colonic contents of pigs at 41 d of age.

Item	Dietary Treatments ^3^					SEM ^1^	*p*-Value ^2^
	SDP	BSF	FM	PCM	HCP		
Duodenum							
Villus height, μm	391.3	403.6	406.8	380.0	420.0	21.379	0.728
Crypt depth, μm	257.6	224.0	235.1	233.1	229.7	11.450	0.303
Villus:crypt ratio	1.52	1.83	1.74	1.66	1.84	0.093	0.102
Jejunum							
Villus height, μm	333.9	353.2	398.4	379.0	361.6	23.630	0.374
Crypt depth, μm	224.4 ^A^	186.7 ^B^	218.1 ^AB^	202.8 ^AB^	198.0 ^AB^	10.248	0.083
Villus:crypt ratio	1.51	1.91	1.85	1.91	1.90	0.150	0.274
pH	5.92	5.87	5.95	5.92	5.87	0.110	0.983
Colon pH							
pH	5.86	5.68	5.83	5.77	5.77	0.085	0.617

^A,B^ Uppercase letters were used to indicate a trend for 0.05 < *p* < 0.10. ^1^ Pooled standard error of the mean; *n* = 9 pigs per treatment. ^2^ *p*-value referring to ANOVA. ^3^ Dietary treatment: SDP, a control diet containing 40 g/kg spray-dried blood plasma; BSF, in which 20 g/kg of SDP was replaced by 20 g/kg black soldier fly larval meal; FM, in which 20 g/kg of SDP was replaced by 20 g/kg fish meal; PCM, in which 20 g/kg of SDP was replaced by 20 g/kg pork crackling meal; HCP, in which 20 g/kg of SDP was replaced by 20 g/kg hydrolyzed chicken protein.

**Table 8:** Effect of insect- and animal-derived protein ingredients on the jejunal mRNA relative abundance of inflammatory, antioxidant, nutrient transporter markers and tight junction proteins of pigs at 41 d of age.

Item ^4^	Dietary Treatments ^3^					SEM ^1^	*p*-Value ^2^
	SDP	BSF	FM	PCM	HCP		
*CAT*	1.00	0.83	0.65	0.58	0.92	0.140	0.641
*GPX*	1.00	0.77	0.45	0.37	0.94	0.199	0.160
*SOD*	1.00	0.98	0.56	0.68	1.00	0.142	0.342
*ZO1*	1.00 ^a^	0.56 ^ab^	0.34 ^b^	0.31 ^b^	0.61 ^ab^	0.144	0.049
*OCL*	1.00	0.50	0.44	0.44	0.78	0.128	0.379
*EAAC1*	1.00	0.66	0.59	0.68	0.99	0.188	0.797
*GLUT2*	1.00	1.13	0.71	0.76	1.24	0.136	0.561
*IL-10*	1.00	1.24	0.61	0.58	1.37	0.215	0.200
*IFN-* *γ*	1.00	1.10	0.63	0.80	1.49	0.220	0.407
*TNF-α*	1.00 ^a^	0.29 ^bc^	0.36 ^ab^	0.18 ^c^	0.65 ^ab^	0.156	0.022
*MCT1*	1.00	0.53	0.38	0.48	0.97	0.194	0.119
*PepT1*	1.00 ^A^	0.46 ^AB^	0.32 ^B^	0.40 ^AB^	0.60 ^AB^	0.116	0.052
*SGLT1*	1.00	0.42	0.29	0.19	0.66	0.127	0.247
*y+LAT1*	1.00	0.67	0.46	0.51	0.83	0.107	0.220

^a,b,c^ Means followed by different lowercase letters differ statistically at the *p* ≤ 0.05 level, while uppercase letters were used to indicate a trend for 0.05 < *p* < 0.10. ^1^ Pooled standard error of the mean; *n* = 9 pigs per treatment. ^2^ *p*-value referring to ANOVA. ^3^ Dietary treatment: SDP, a control diet containing 40 g/kg spray-dried blood plasma; BSF, in which 20 g/kg of SDP was replaced by 20 g/kg black soldier fly larval meal; FM, in which 20 g/kg of SDP was replaced by 20 g/kg fish meal; PCM, in which 20 g/kg of SDP was replaced by 20 g/kg pork crackling meal; HCP, in which 20 g/kg of SDP was replaced by 20 g/kg hydrolyzed chicken protein. ^4^ *CAT* = catalase; *GPX* = glutathione peroxidase; *SOD* = superoxide dismutase; *ZO1* = zonula occludens 1; *OCL* = occludin; *EAAC1* = excitatory amino acid carrier 1; *GLUT2* = glucose transporter type 2; *IL-10* = interleukin 10; *IFN-γ* = interferon gamma; *TNF-α* = tumor necrosis factor alpha; *PepT1* = peptide transporter 1; *SGLT1* = Na +/glucose co-transporter 1; *MCT1* = monocarboxylate transporter 1; *y+LAT1* = Na+-independent cationic and Na+-dependent neutral amino acid transporter 1.

## Data Availability

The data presented in this study are available on request from the corresponding author.
